# Intracellular Chloride Regulation in AVP+ and VIP+ Neurons of the Suprachiasmatic Nucleus

**DOI:** 10.1038/s41598-017-09778-x

**Published:** 2017-08-31

**Authors:** Nathan J. Klett, Charles N. Allen

**Affiliations:** 1 Neuroscience Graduate Program, School of Medicine, Oregon Health & Science University, Portland, OR 97239 USA; 2 Oregon Institute for Occupational Health Sciences, School of Medicine, Oregon Health & Science University, Portland, OR 97239 USA; 3 Department of Behavioral Neuroscience, School of Medicine, Oregon Health & Science University, Portland, OR 97239 USA

## Abstract

Several reports have described excitatory GABA transmission in the suprachiasmatic nucleus (SCN), the master pacemaker of circadian physiology. However, there is disagreement regarding the prevalence, timing, and neuronal location of excitatory GABA transmission in the SCN. Whether GABA is inhibitory or excitatory depends, in part, on the intracellular concentration of chloride ([Cl^−^]_i_). Here, using ratiometric Cl^−^ imaging, we have investigated intracellular chloride regulation in AVP and VIP-expressing SCN neurons and found evidence suggesting that [Cl^−^]_i_ is higher during the day than during the night in both AVP+ and VIP+ neurons. We then investigated the contribution of the cation chloride cotransporters to setting [Cl^−^]_i_ in these SCN neurons and found that the chloride uptake transporter NKCC1 contributes to [Cl^−^]_i_ regulation in SCN neurons, but that the KCCs are the primary regulators of [Cl^−^]_i_ in SCN neurons. Interestingly, we observed that [Cl^−^]_i_ is differentially regulated between AVP+ and VIP+ neurons-a low concentration of the loop diuretic bumetanide had differential effects on AVP+ and VIP+ neurons, while blocking the KCCs with VU0240551 had a larger effect on VIP+ neurons compared to AVP+ neurons.

## Introduction

The suprachiasmatic nucleus (SCN) of the anterior hypothalamus is the master pacemaker of the circadian system. Besides a cohort of neuropeptides, SCN neurons synthesize and package the neurotransmitter GABA. GABA transmission regulates synaptic input from the RHT^1^, mediates phase shifts^2, 3^, regulates firing frequency^4^, and contributes to circadian synchrony^5–9^.

GABA is the primary inhibitory neurotransmitter in the central nervous system, but has been observed to be excitatory during embryonic and neonatal development, in certain pathologies, as well as in several areas of the adult brain^10–12^. Interestingly, excitatory GABA transmission has been observed in the mature SCN^6, 7, 13–20^. However, reports have disagreed on the prevalence, timing, and neuronal location of excitatory GABA transmission. GABA has been reported to be exclusively inhibitory, inhibitory during the day and excitatory during the night, and excitatory during the night and inhibitory during the day^7, 13–15, 17, 20–22^. Additionally, the proportion of neurons within the SCN neural network that are excited by GABA may be involved in encoding day length^18, 23^.

The GABA_A_ receptor is permeable to both Cl^−^ and HCO_3_^−^ ions with a relative permeability ratio of approximately 0.8^24–26^. Because the GABA_A_ receptor is primarily permeable to Cl^−^ ions, whether GABA is depolarizing or hyperpolarizing depends on the intracellular concentration of chloride ([Cl^−^]_i_) and the membrane potential. [Cl^−^]_i_ is regulated by a family of cation chloride cotransporters (CCCs) which use the concentration gradients of Na^+^ and K^+^ ions to transport Cl^−^ ions into (the sodium-potassium-chloride cotransporter 1, NKCC1) or out of (the potassium-chloride cotransporters, KCC) neurons. Normally, [Cl^−^]_i_ is kept low in neurons by the action of the neuron-specific^27, 28^ isotonically-active^29–31^ KCC2. However, a role for NKCC1 in [Cl^−^]_i_ regulation has been demonstrated in the SCN—blocking NKCC1 with bumetanide decreased the amplitude of GABA-induced Ca^2+^ transients^15, 16, 18^ and hyperpolarized the GABAergic reversal potential^15, 17^.

Interestingly, immunohistochemistry has revealed differential expression of chloride transporters throughout the SCN^32^. KCC2 expression was most dense in the ventrolateral SCN, and correlated with vasoactive intestinal peptide (VIP) expression. Alternatively, KCC3 and KCC4 expression was concentrated in the dorsomedial SCN. NKCC1 was expressed throughout the SCN, but was concentrated in the dorsomedial SCN, and correlated with vasopressin (AVP) expression. The differential expression of the CCCs throughout the SCN suggests that [Cl^−^]_i_ and the GABAergic equilibrium potential (E_GABA_) may vary regionally throughout the SCN. Indeed, Albus *et al*. observed that GABA is exclusively inhibitory in the ventral SCN, but is excitatory in the dorsal SCN during the late day and early night^6^. Similarly, GABA-induced Ca^2+^ transients were found to be most prevalent in the dorsomedial SCN^16^, and using the Cl^−^-sensitive dye MQAE, [Cl^−^]_i_ was found to be elevated in the dorsomedial SCN^9^. Therefore, there is mounting evidence to support the idea that subpopulations of SCN neurons differ in their regulation of the intracellular chloride concentration.

In this work, we used epifluorescent imaging of a genetically-encoded chloride indicator to examine regional and circadian regulation of [Cl^−^]_i_ in the SCN. Cl-Sensor is a ratiometric chloride indicator composed of a CFP moiety linked to a YFP moiety whose emission is sensitive to chloride in a physiological range (YFP_Cl_). Ratiometric Cl^−^ imaging allows estimation of [Cl^−^]_i_ from multiple cells simultaneously without disrupting the native cellular milieu. Using a transgenic strategy, we targeted Cl-Sensor to AVP or VIP-expressing SCN neurons, and were able to detect both transient and persistent changes in [Cl^−^]_i_.

Our results indicate that GABA_A_ receptor activation elicits Cl^−^ influx in AVP+ and VIP+ neurons in the SCN. Accordingly, we show that the KCCs play a major role in [Cl^−^]_i_ regulation in SCN neurons, while the chloride importer NKCC1 has a relatively minor role in setting resting [Cl^−^]_i_. Further, we show that [Cl^−^]_i_ is differentially regulated in AVP and VIP-expressing neurons.

## Results

**Figure 1:**
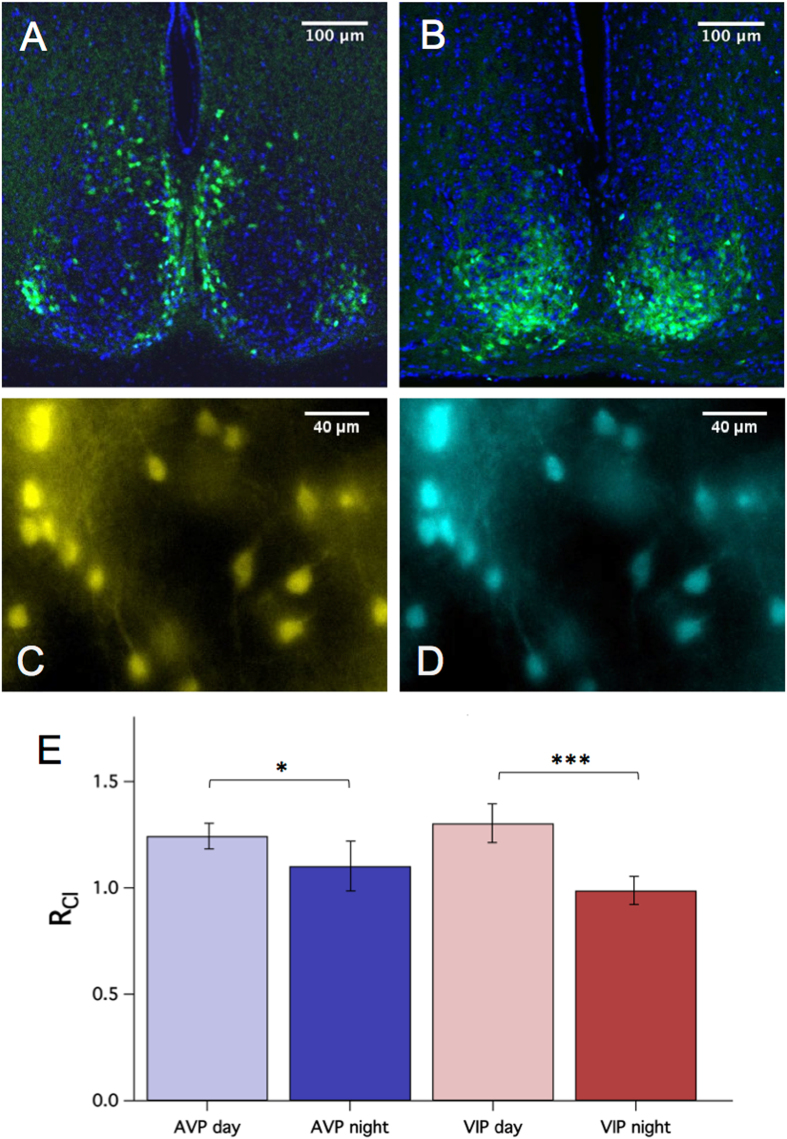
R_Cl_ measurement in genetically-identified SCN neurons. Confocal micrographs demonstrating regional expression of Cl-Sensor in the SCN of AVP::Cl-Sensor (**A**) and VIP::Cl-Sensor (**B**) mice. Native Cl-Sensor fluorescence is depicted in green, and DAPI stain is shown in blue. Pseudocolored epifluorescent micrographs of an AVP::Cl-Sensor SCN slice exhibiting YFP (**C**) and CFP (**D)** emission. (**E**) Baseline R_Cl_ was higher during the day (ZT 2 to 8) compared to the night (ZT 12 to 18) for both AVP+ (GEE, *p* < 0.05) and VIP+ (GEE, *p* < 0.001) neurons.

Vasoactive intestinal peptide (VIP) and vasopressin (AVP) mark the “core” and “shell” partition that has served as a useful anatomical model for dissecting SCN function. Generally, sensory inputs project to the ventrolateral SCN core, while core neurons synapse unto neurons in the dorsomedial shell. Several reports have suggested that excitatory GABA transmission may correlate with this anatomical feature^13–15, 17, 20^. Therefore, we performed Cl^−^ imaging in VIP+ and AVP+ neurons of the SCN to look for differences in [Cl^−^]_i_ regulation between these two populations of neurons. To measure [Cl^−^]_i_ in SCN neurons, we used a newly-developed Cre-inducible mouse line, with a floxed Cl-Sensor allele inserted into the Rosa26 locus^33^. To obtain Cl-Sensor expression in the SCN, we crossed these mice with either VIP-IRES-Cre mice or AVP-IRES-Cre mice to give Cl-Sensor expression in either VIP+ or AVP+ neurons^34, 35^. The resultant VIP::Cl-Sensor mice displayed Cl-Sensor expression in the ventrolateral SCN, while the AVP::Cl-Sensor mice displayed Cl-Sensor expression in the dorsomedial SCN, as expected for VIP and AVP expression (Fig. 1A,B ^36^).

Several studies have indicated that excitatory GABA transmission demonstrates circadian rhythmicity^13, 14, 16, 17, 20^. Therefore, we first compared baseline values of R_Cl_ during the day (ZT 2 to 8) and night (ZT 12 to 18) in AVP+ and VIP+ SCN neurons. Interestingly, R_Cl_ was higher during the day in both AVP+ (generalized estimating equations (GEE), *p* < 0.05) and VIP+ (GEE, *p* < 0.001) neurons (Fig. 1E) suggesting that [Cl^−^]_i_ is higher during the day in these neurons. Several reports have described regional variability of excitatory GABA transmission, with some agreement that it is more common in the dorsal SCN, suggesting that this phenomenon may be specific to AVP+ neurons. Indeed, in the nearby paraventricular nucleus, Haam *et al*. observed that AVP+ neurons had a more depolarized E_GABA_ relative to their AVP− neighbors^37^. To determine whether AVP+ neurons demonstrate higher [Cl^−^]_i_ relative to VIP+ neurons, we compared baseline R_Cl_ values between VIP+ and AVP+ neurons, but found no significant difference in R_Cl_ during either the day or the night (Fig. 1E).

**Figure 2:**
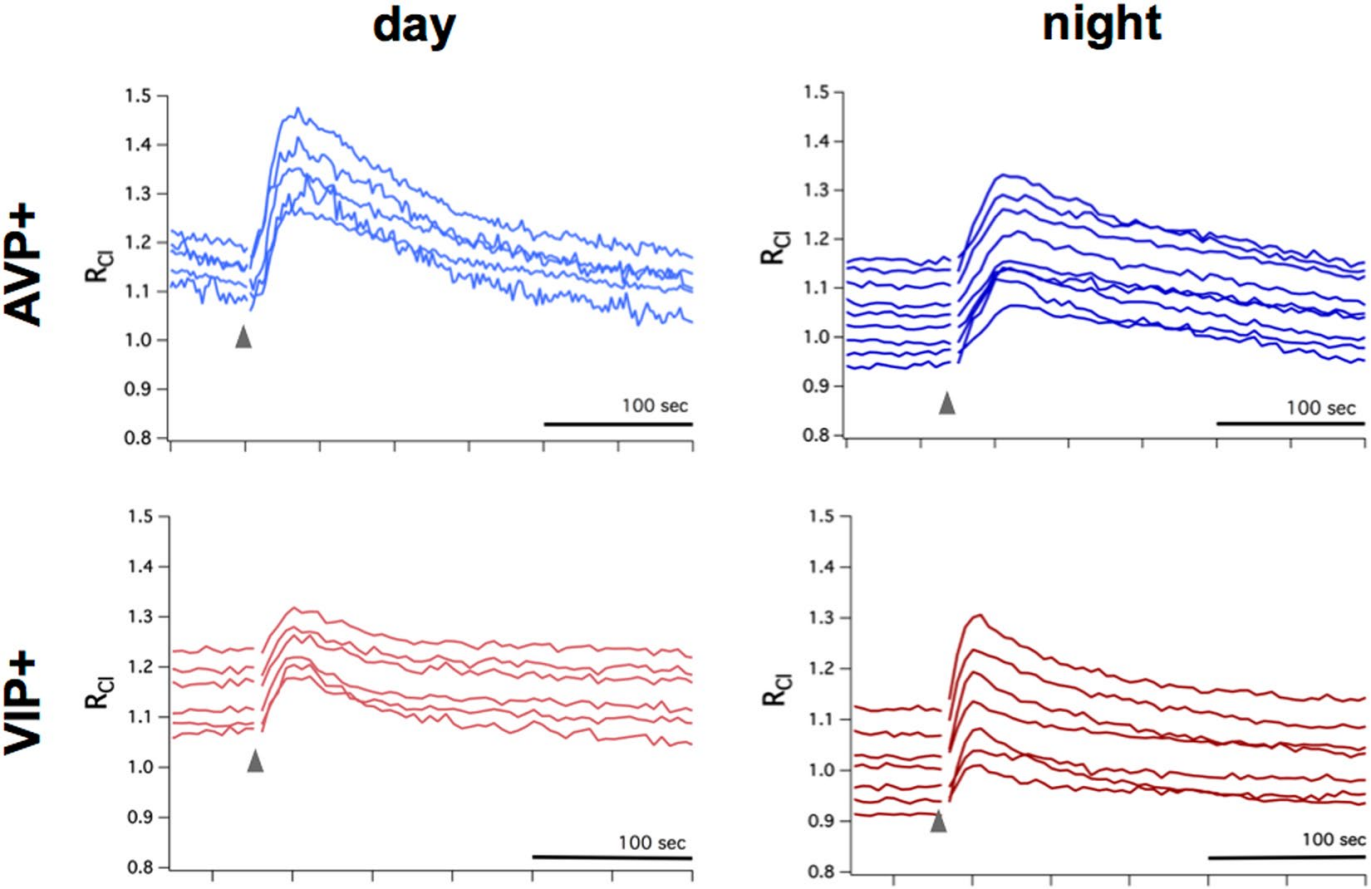
GABA_A_ receptor-mediated Cl^−^ transients in AVP+ and VIP+ neurons. Top row: AVP+ neurons from AVP::Cl-Sensor mice tested during subjective day (left) and subjective night (right) demonstrated an increase of R_Cl_ following puff application of the GABA_A_ agonist isoguvacine (gray arrow), indicative of inhibitory Cl^−^ influx. Similarly, VIP+ neurons from VIP::Cl-Sensor mice during both day and night responded to isoguvacine with an increase of R_Cl_ (bottom row). Each trace represents a R_Cl_ measurement obtained from a single neuronal soma.

A hyperpolarizing action of GABA will elicit Cl^−^ influx, while a depolarizing action will elicit Cl^−^ efflux. To assess the polarity of GABA transmission in SCN neurons, we performed puff applications of the GABA_A_ agonist isoguvacine in the presence of 2 µM TTX to block potential polysynaptic effects. In response, R_Cl_ increased in AVP+ neurons during the day and night, indicative of Cl^−^ influx and inhibitory GABA transmission (Fig. 2, top row). Cl^−^ influx was also observed in VIP+ neurons during both the day and the night (Fig. 2, bottom row). These results indicate that GABA is inhibitory in both AVP+ and VIP+ SCN neurons. Interestingly, these GABA_A_-induced Cl^−^ transients lasted for minutes, much longer than expected for GABA_A_-induced currents, but similar to the timecourse of Cl^−^ transients reported in other cells^38–40^. The timecourse of these Cl^−^ transients may represent the activity of chloride transporters. Indeed, it has been shown that transient shifts in GABA polarity can last for minutes and that chloride transporters mediate this equilibration process^41–43^.

**Figure 3:**
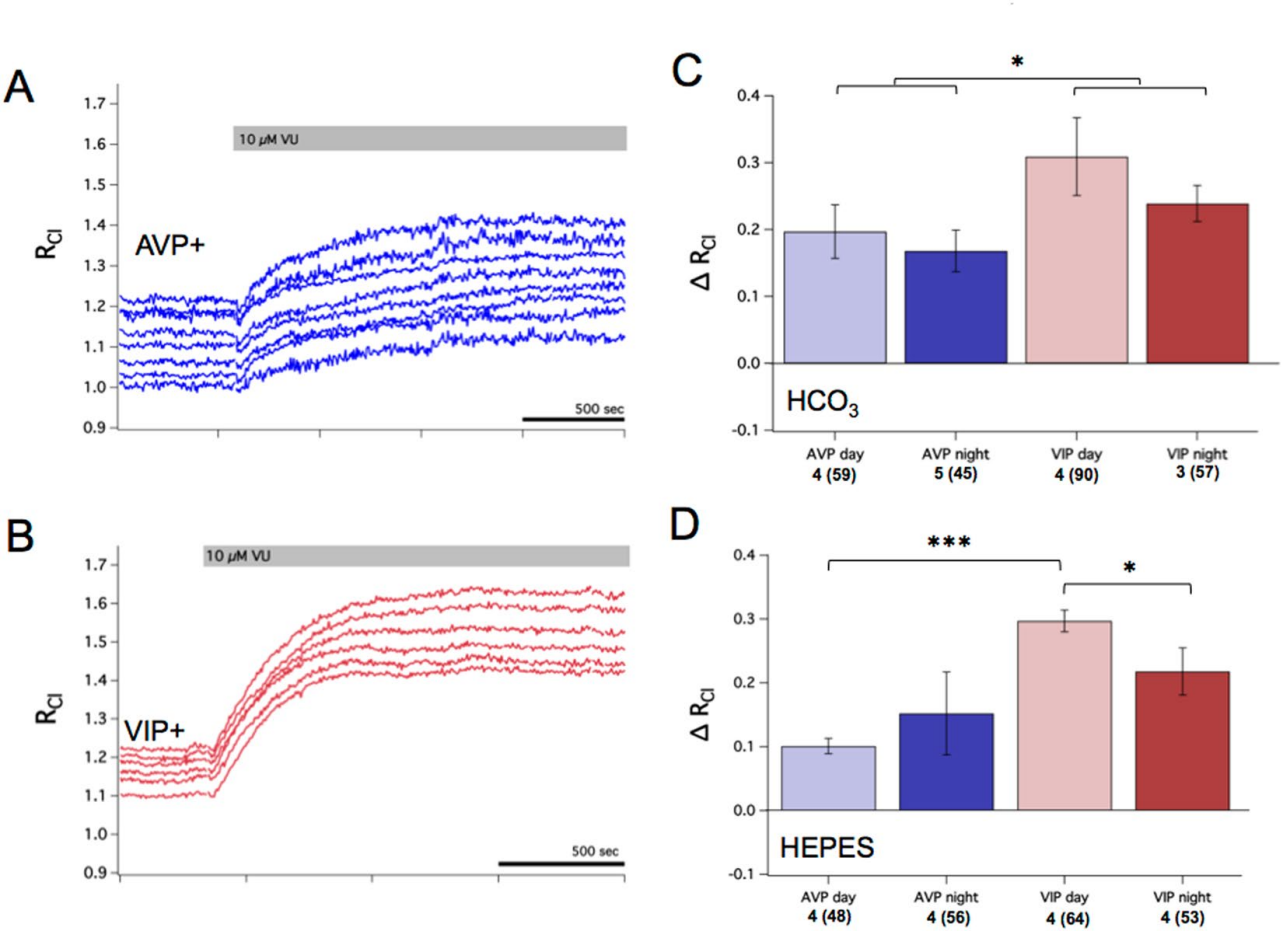
The KCCs regulate [Cl^−^]_i_ in SCN neurons. (**A**) Example experiment from an AVP::Cl-Sensor mouse recorded during the night demonstrating the effect of 10 µM of the KCC antagonist VU. VU caused an increase in R_Cl_ indicative of a rise in [Cl^−^]_i_. Each trace represents a R_Cl_ measurement obtained from a single neuronal soma. (**B**) Example experiment from a VIP::Cl-Sensor mouse recorded during the day demonstrating the effect of VU. VU caused an increase in R_Cl_ indicative of a rise in [Cl^−^]_i_. (**C**) Summary data of the average change in R_Cl_ after VU by neuron type and time of day. VU resulted in an increase in R_Cl_ in all conditions (GEE, *p* < 0.005), but had a significantly greater effect in VIP+ neurons compared to AVP+ neurons (GEE, *p* < 0.05). (**D**) Summary of changes in R_Cl_ elicited by VU in a HEPES-buffered solution. VU had a larger effect during the day in VIP+ neurons when compared to VIP+ night (GEE, *p* < 0.05) and AVP+ day neurons (GEE, *p* < 0.001). For (**C**) and (**D**), the number of slices and total regions of interest (in parentheses) is listed for each condition under the x-axis.

Therefore, we next examined the degree to which the CCCs set [Cl^−^]_i_ in SCN neurons. Since our GABA_A_-induced Cl^−^ transients indicated inhibitory Cl^−^ influx upon GABA_A_ receptor activation, we first investigated the contribution of KCC2, the neuron-specific CCC responsible for keeping [Cl^−^]_i_ levels low in neurons throughout the brain. To test for the activity of KCC2 in setting resting [Cl^−^]_i_ we used VU0240551, an antagonist that selectively targets the KCCs^44^. VU increased R_Cl_ by 0.18 in AVP+ and by 0.27 in VIP+ neurons (Fig. 3A,B). Based on our calibration curve (Supplementary Fig. 1), we estimate these changes in R_Cl_ to reflect a 15 mM increase in [Cl^−^]_i_ in AVP+ neurons and a 29 mM increase in VIP+ neurons. VU had a significantly greater effect in VIP+ neurons compared to AVP+ neurons (GEE, *p* < 0.05), but there were no day/night differences within neuron type (Fig. 3C). Because several Cl^−^ transporters are also transporters for the bicarbonate ion, we wondered if removing bicarbonate ions from the extracellular solution might alter [Cl^−^]_i_ and therefore the efficacy of VU. Therefore, we repeated VU application in a separate set of experiments using a HEPES-buffered solution (Fig. 3D). Blocking the KCCs with VU gave a pattern of results similar to that observed with solution containing bicarbonate. VU elicited an increase in R_Cl_ in all conditions, indicative of [Cl^−^]_i_ increase. When comparing the effect of VU across solutions, we observed a difference in the amplitude of VU’s effect in AVP+ neurons during the day (two-sample *z*-test, *p* < 0.05). Furthermore, experiments in HEPES solution revealed a day/night difference in the effect of VU that was not present in the bicarbonate solution (Fig. 3D, GEE, *p* < 0.05). In a separate set of experiments, we examined the effect of VU on the GABAergic reversal potential (E_GABA_) using perforated-patch recording. In these experiments, VU elicited a depolarization of E_GABA_ by approximately 23 mV (paired *t*-test, *p* < 0.001), and slowed the recovery of resting E_GABA_ after Cl^−^ loading (paired *t*-test, *p* < 0.05) (see Supplementary Fig. 2). Collectively, we interpret these findings to indicate that the KCC family of chloride cotransporters play a major role in [Cl^−^]_i_ regulation in SCN neurons.

**Figure 4:**
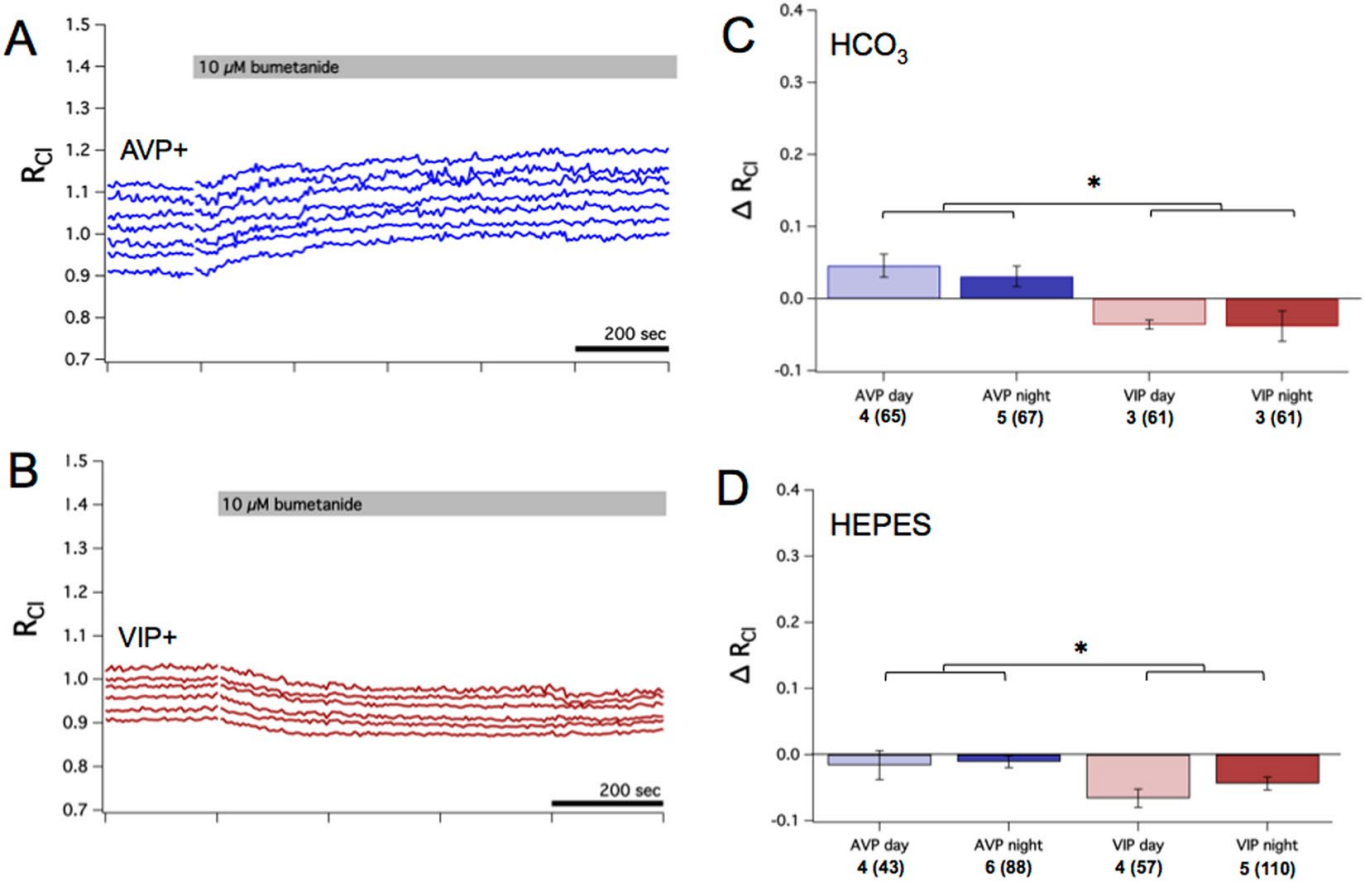
NKCC1 contributes to setting resting [Cl^−^]_i_ in SCN neurons. (**A**) Example experiment from an AVP::Cl-Sensor mouse recorded during the night showing the effect of blocking NKCC1 with 10 µM of bumetanide. Bumetanide caused a small increase in R_Cl_. Each trace represents a R_Cl_ measurement obtained from a single neuronal soma. (**B**) Example experiment from a VIP::Cl-Sensor mouse recorded during the night demonstrating the effect of bumetanide. Bumetanide caused a small decrease in R_Cl_. (**C**) Summary data of the average change in R_Cl_ after bumetanide by neuron type and time of day. Bumetanide elicited small but statistically significant changes in R_Cl_ in each condition (GEE, *p* < 0.005). AVP+ and VIP+ neurons responded differently to bumetanide (GEE, *p* < 0.001), but there were no day/night differences within neuron types. Bumetanide had a statistically different effect on VIP+ neurons compared to AVP+ neurons. (**D**) Summary of changes in R_Cl_ elicited by bumetanide in a HEPES-buffered solution. Bumetanide had a larger effect in AVP+ neurons compared to VIP+ neurons (GEE, *p* < 0.001). For (**C**) and (**D**), the number of slices and total regions of interest (in parentheses) is listed for each condition under the x-axis.

Previous studies have implicated NKCC1 in [Cl^−^]_i_ regulation in SCN neurons^15–18, 32^. To test for a contribution of NKCC1 to resting [Cl^−^]_i_, we used the loop diuretic bumetanide which selectively targets NKCC1 when used at 10 µM^45^. Bumetanide increased R_Cl_ in AVP+ neurons by approximately 0.04 (~4 mM) and decreased R_Cl_ in VIP+ neurons by 0.04 (~3 mM) (Fig. 4). These changes were small but significantly different from baseline (GEE, *p* < 0.005). As with VU, we observed differences in the effect of bumetanide between AVP+ and VIP+ neurons (GEE, *p* < 0.001), but no day/night differences within neuron type. Surprisingly, bumetanide elicited a small increase in [Cl^−^]_i_ in AVP+ neurons, contrary to its expected role in blocking Cl^−^ uptake. This result may be due to off-target effects of bumetanide, which is known to inhibit the KCCs at higher concentrations^10, 44^. In a separate series of experiments, we tested the efficacy of bumetanide in a HEPES-buffered solution, which should diminish Cl^−^/HCO_3_^−^ exchange. In HEPES, bumetanide reduced R_Cl_ in VIP+ neurons but had little effect on AVP+ neurons (Fig. 4D). However, the effect of bumetanide on AVP+ neurons was significantly greater in the bicarbonate-buffered solution compared to the HEPES-buffered solution (two-sample *z*-test, *p* < 0.05). As in the bicarbonate-buffered experiments, bumetanide elicited a greater effect in VIP+ neurons compared to AVP+ neurons (GEE, *p* < 0.05). Using perforated-patch recording, we also investigated the effect of bumetanide on E_GABA_. In these experiments, bumetanide did not significantly alter E_GABA_ or the timecourse for the recovery of E_GABA_ following a Cl^−^ depletion protocol (Supplementary Fig. 3). Overall, we observed relatively small effects of bumetanide compared to VU, suggesting that the KCCs are the major regulators of resting [Cl^−^]_i_ in SCN neurons.

**Figure 5:**
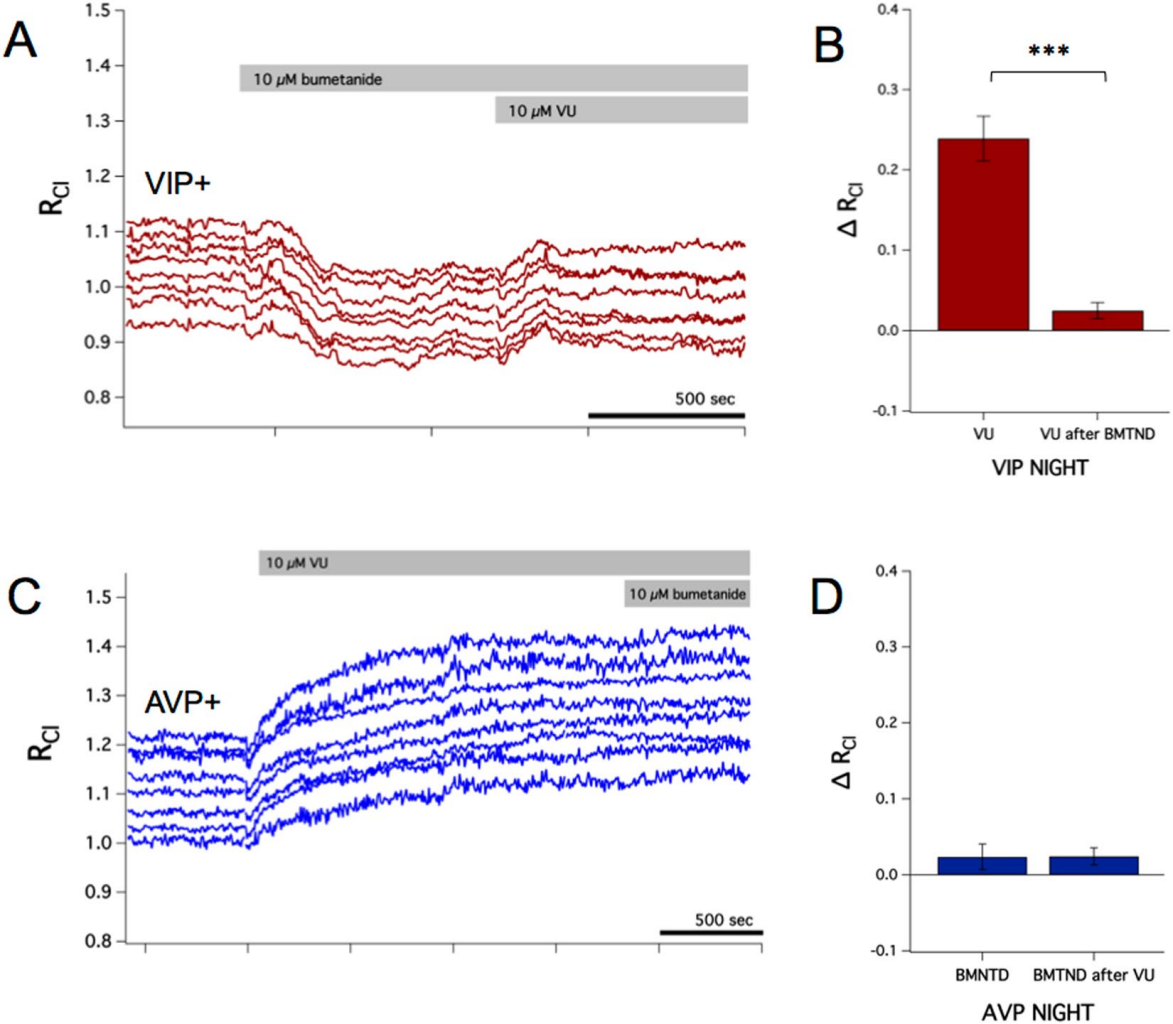
Bumetanide occludes the effect of VU. (**A**) Example experiment from a VIP::Cl-Sensor mouse recorded during the night in which VU (10 µM) was applied after bumetanide (10 µM). From rest, VU elicits a 0.24 increase in R_Cl_ on average (**B**, same data from Fig. 3). However, the effect of VU was occluded in the presence of bumetanide (GEE, *p* < 0.001), suggesting that NKCC1 mediates the Cl^−^ accumulation elicited by VU. Conversely, the effect of bumetanide in the presence of VU was similar to the effect of bumetanide alone (Fig. C and D), indicating that the KCCs are necessary to mediate Cl^−^ extrusion in these neurons.

The previous results indicate that both NKCC1 and the KCC family of cotransporters contribute to resting [Cl^−^]_i_ in SCN neurons. We next investigated how these cotransporters interact to regulate [Cl^−^]_i_. As discussed, blocking the KCCs with VU resulted in a substantial increase in R_Cl_ (Fig. 3). However, because of the relatively minor effect of bumetanide, it was not clear what Cl^−^ uptake pathways were mediating the effect of VU. In order to determine if NKCC1 mediates Cl^−^ uptake in the absence of the KCCs, we applied VU after bumetanide treatment (Fig. 5A). The effect of VU was largely occluded in the presence of bumetanide (Fig. 5A,B). Therefore, although NKCC1 has a relatively minor role in setting resting [Cl^−^]_i_, it does constitute a tonic Cl^−^ influx pathway in SCN neurons. Conversely, we next examined whether blocking the KCCs could reveal a greater bumetanide effect. However, the amplitude of bumetanide’s effect was similar in the presence or absence of VU (Fig. 5C,D), indicating that the KCCs are necessary for Cl^−^ extrusion.

## Discussion

We performed somatic Cl^−^ imaging to investigate [Cl^−^]_i_ in two genetically-defined subpopulations of SCN neurons. We found that R_Cl_ was higher in both AVP+ and VIP+ neurons during the day (ZT 2 to 8) compared to the night (ZT 12 to 18), suggesting that [Cl^−^]_i_ is elevated during the day. This observation is in agreement with several reports that have observed increased excitatory GABA transmission during the day and early night^6, 13, 15–17^. However, we observed Cl^−^ influx in response to GABA_A_ receptor activation, indicative of an inhibitory effect of GABA. Similarly, we observed large changes in R_Cl_ after application of the KCC antagonist VU, but small changes after blocking NKCC1 with bumetanide. VU increased [Cl^−^]_i_ dramatically, in the range of 15 to 30 mM, suggesting that the KCCs are the major determinants of [Cl^−^]_i_ in AVP+ and VIP+ SCN neurons. Therefore, our results add to a group of studies that have concluded that GABA is exclusively inhibitory in the mature SCN^5, 46–49^. Still, it should be remembered that the SCN is a very heterogeneous nuclei and that AVP and VIP-expressing neurons only constitute ~13% and ~9% of all SCN neurons respectively^50, 51^, leaving open the possibility that our study did not address the SCN neurons demonstrating excitatory GABA.

The descriptions of excitatory GABA transmission in the SCN have been riddled with discrepancies. Differences in methodology are likely to underlie some of these inconsistencies. Indeed, whole-cell, perforated-patch, cell-attached, and multi-unit recording techniques as well as Ca^2+^ imaging have all been used to address the polarity of GABA transmission in the SCN. The timing of inhibitory post-synaptic currents within the interspike interval is critical in determining whether inhibitory currents will speed up or slow cell firing, further highlighting the nuance of GABA transmission in the SCN^4, 9, 52^. The issue may also be related to the complexity of intracellular Cl^−^ regulation itself. Besides neurotransmission, [Cl^−^]_i_ is an important cellular feature linked to processes such as pH regulation, cell volume regulation, and even membrane potential^12, 43, 54^. Therefore, cell turgidity as well as the osmolarity and pH of solutions are all likely to influence measures of [Cl^−^]_i._ Further, [Cl^−^]_i_ has been shown to change after neuronal damage, and in relation to the proximity of cells to the surface of a brain slice^55, 56^. Additionally, previous studies have not adequately ruled out the possibility that disinhibition underlies the observed excitatory effects of GABA transmission in the SCN. The SCN network is known to have diffuse local connectivity^7, 19, 57^. Therefore, polysynaptic effects must be considered when applying GABA agonists to SCN neurons. Without the inclusion of TTX in the recording media, GABA-mediated inhibition of pre-synaptic inputs could be read out as excitation in the cell of interest. Further, some of the data in support of excitatory GABA transmission has been inferred by the effects of the GABA_A_ receptor antagonist bicuculline^14, 15, 58^. Regrettably, these results are confounded by the off-target effects of bicuculline, which is known to antagonize SK channels at commonly used concentrations^59^. Indeed, SK channels have been shown to contribute to the resting membrane potential, afterhyperpolarization and spike-frequency adaptation of SCN neurons^60, 61^.

We have successfully performed Cl^−^ imaging techniques in SCN neurons, and have demonstrated their utility for monitoring Cl^−^ flux and [Cl^−^]_i_ regulation. Cl^−^ imaging offers several advantages compared to gramicidin perforated patch recording by leaving V_m_ unperturbed and by allowing for the sampling of multiple neurons simultaneously. Further, the use of a genetically-encoded indicator allowed us to target specific populations of SCN neurons. Nevertheless, Cl-Sensor has room for improvement. Cl-Sensor’s sensitivity to Cl^−^ is not optimal for normal neuronal concentrations of chloride, and the intrinsic H^+^ sensitivity of the indicator within a physiological pH range can be problematic.

Our methodology did not allow us to investigate subcellular differences in R_Cl_. Indeed, intracellular Cl^−^ gradients have been reported in several types of neurons throughout the brain (see^62^ for review). For example, a two-photon Cl^−^ imaging study observed a somatodendritic chloride gradient in a class of retinal bipolar cells, concluding that Cl^−^ is 20 mM higher in dendrites relative to the soma^63^. A somatodendritic Cl^−^ gradient could explain why previous studies have shown GABA-evoked Ca^2+^ transients in SCN neurons^15, 16, 18^, supporting an excitatory role of GABA, while we have observed inhibitory Cl^−^ influx. While dendritic depolarization may be able to activate somatic voltage-gated calcium channels, Cl^−^ efflux at dendrites may not be registered by somatic Cl^−^ imaging. Similarly, a somatodendritic Cl^−^ gradient could explain why bumetanide was able to diminish GABA-evoked Ca^2+^ transients, but produced small changes in our measurements of somatic R_Cl_. Higher-resolution imaging techniques will be necessary to address whether somatodendritic Cl^−^ gradients exist in SCN neurons.

Recently, the role of the CCC’s in determining [Cl^−^]_i_ has come under scrutiny by compelling two-photon Cl^−^ imaging results which argue for the primacy of large intracellular anions in setting [Cl^−^]_i_^64^. In their study, Glycks *et al*. observed little effect of blocking either NKCC1 or KCC2 in hippocampal and neocortical pyramidal neurons, while we found a small effect of bumetanide and a clear effect of VU. Neuron type could underlie this incongruity. Alternatively, while we monitored R_Cl_ continuously, Glycks *et al*. sampled R_Cl_ before and 30 minutes after application of bumetanide and VU, leaving open the possibility that secondary homeostatic mechanisms reset [Cl^−^]_i_ during that time.

Although we did not observe a difference in resting R_Cl_ between AVP+ and VIP+ neurons, we did observe differential regulation of [Cl^−^]_i_ between AVP+ and VIP+ neurons. We found that AVP+ and VIP+ neurons differed in their sensitivity to both VU and bumetanide. The increased sensitivity of VIP+ neurons to VU suggests that they may have lower resting [Cl^−^]_i_ relative to AVP+ neurons, consistent with studies that have observed a greater prevalence of excitatory GABA transmission in the dorsal SCN^6, 15, 16^ as well as with recent data from two groups who, using the Cl^−^ sensitive dye MQAE, concluded that [Cl^−^]_i_ is elevated in the dorsal SCN^9, 23^. Previous *in situ* hybridization^65^ and immunocytochemical^66^ studies have described regional expression of chloride transporters in the rat SCN. Therefore, these regional differences in expression may explain the differential effects of VU and bumetanide in AVP+ and VIP+ neurons. Specifically, KCC2 expression was limited to the ventrolateral SCN, and colocalized with neurons containing GRP or VIP. Markedly, KCC2 expression was absent from the dorsomedial SCN and did not colocalize with AVP—rather, KCC3 and KCC4 were found in the dorsomedial SCN^32^. This histology is in agreement with our observation that VU had a larger effect in VIP+ neurons compared to AVP+ neurons. Despite the paucity of KCC2 in the dorsomedial SCN, we observed that VU increased [Cl^−^]_i_ in AVP+ neurons, albeit less than it did in VIP+ neurons. The efficacy of VU in the AVP+ neurons may be explained by the non-specificity of VU for KCC2^44^. VU may have acted on KCC3 or KCC4 in AVP+ neurons.

Generally, resting membrane potential in SCN neurons is approximately −45 mV during the night and exhibits oscillations of roughly 10 to 15 mV throughout the day^61, 67^. For a neuron with a V_m_ of −45 mV, [Cl^−^]_i_ is passively distributed at approximately 15 mM. Therefore, our estimates of resting [Cl^−^]_i_ (greater than 20 mM) indicate that there are constitutively-active uptake mechanisms in SCN neurons. In VIP+ neurons, blocking NKCC1 decreased [Cl^−^]_i_, supporting a role for NKCC1-mediated Cl^−^ uptake in setting resting [Cl^−^]_i_. However, in AVP+ neurons, bumetanide caused a small increase in [Cl^−^]_i_, suggesting that other uptake mechanisms are active in AVP+ neurons. Accordingly, Choi *et al*. observed that GABA-induced Ca^2+^ transients remained in NKCC1 knockout mice^15^. This Cl^−^ uptake may be mediated by the anion exchangers (AEs), which exchange intracellular bicarbonate for extracellular chloride, or could be mediated by a Cl^−^ channel^12, 43^. Indeed, our results in HEPES-buffered solutions, which minimize the presence of bicarbonate transport, imply the presence of bicarbonate-dependent Cl^−^ regulation in SCN neurons. Although generally similar, the experiments done in HEPES-buffered solution revealed several interesting differences. The effect of bumetanide in AVP+ neurons differed between solutions, and experiments done in HEPES-buffered solution revealed a day/night difference in VU’s effect on VIP+ neurons (Figs 3 and 4). These findings implicate the activity of the Na^+^-driven Cl-HCO_3_ exchanger (NDCBE) or the AE family of cotransporters in SCN neurons. Further research will be necessary to address the role of these transporters in SCN physiology.

Overall, our results demonstrate day/night and regional differences in [Cl^−^]_i_ regulation and highlight the KCC family of chloride co-transporters as regulators of [Cl^−^]_i_ in SCN neurons. Therefore, our results add to a growing number of studies that point to the importance of [Cl^−^]_i_ in SCN function.

## Methods

### Animal strains and housing

Cl^−^ imaging experiments were performed with C57BL/6 mice in which a floxed Cl-Sensor allele was inserted into the Rosa26 locus^33^. We crossed these mice with either AVP-IRES-Cre^34^ or VIP-IRES-Cre mice^34, 35^ to yield AVP::Cl-Sensor or VIP::Cl-Sensor mice. Tail snips were sent to an external facility for genotyping (Transnetyx, Inc). Mice were heterozygous for both the Cl-Sensor and Cre transgenes. Tissue was prepared from adult male and female mice between two and six months old. Electrophysiological experiments were performed on wild type male Wistar rats aged 3 to 9 months.

All animals were entrained to 12:12 light-dark (LD) cycles, with the time of lights on represented as ZT 0. All procedures were approved in advance by the Institutional Animal Care and Use Committee of Oregon Health and Science University, and all experiments were performed in accordance with the approved animal protocol.

### Confocal microscopy of fixed tissue

Each mouse was deeply anaesthetized with isoflurane and transcardially perfused with 10 mL of phosphate buffered saline (PBS) followed by 10 mL of 4% paraformaldehyde solution in PBS (pH 7.4). The brain was removed and post-fixed for 1-2 hours at 4 °C in the same solution. After repeated washes in 0.1 M PB, the brain was blocked and secured to a vibratome insert with cyanoacrylate adhesive and agarose supports. Coronal (40 µm thick) sections were cut with a Leica vibratome in 0.1 M PB and subsequently washed in the same buffer. For optical clearing, we treated the tissue with a glycerol/0.1 M PB gradient (25% to 90%). The tissue was incubated in each solution at 4 °C with light agitation until equilibrated. After clearing, sections were transferred into a 10% glycerol/0.1 M PB solution and counterstained with DAPI. Tissue sections were transferred onto glass slides in 10% glycerol solution and the cover glass was mounted with ProLong Diamond media after removing the excess buffer. Images were taken with a Zeiss Axioskop 2 TM fluorescent microscope using AxioVision 4.8 software (Carl Zeiss MicroImaging, Inc.). Confocal micrographs consisted of several 0.4 μm thick optical sections adjusted for optimal brightness and contrast using FIJI software.

### Acute slice preparation

During their light phase (ZT 1-3 for day experiments and ZT 10-12 for night experiments), animals were removed from their housing chambers, anaesthetized with isoflurane, and decapitated. The brain was quickly removed and submerged in an ice-cold slicing solution consisting of (in mM): 111 NaCl, 26 NaHCO_3_, 11 dextrose, 6 Na-gluconate, 4 MgCl_2_, 3 KCl, 1 NaH_2_PO_4_, and 0.5 CaCl_2_, saturated with 95% O_2_, 5% CO_2_. The brain was blocked and 175 μm thick coronal slices were prepared with a Leica VT1000S vibrating blade microtome. Slices were incubated in slicing solution for 1-4 hours at 34 **°**C before recording.

### Cl^−^ imaging from acute SCN slices

During image acquisition, slices were perfused at 1–2 mL/min with an artificial cerebrospinal fluid (aCSF) with Cl^−^ adjusted to the physiological concentration (122 mM; ref. 68). aCSF contained (in mM): 114 NaCl, 26 NaHCO_3_, 11 dextrose, 6 Na-gluconate, 2.7 KCl, 2 CaCl_2_, 1 MgCl_2_, and 1 NaH_2_PO_4_, saturated with 95% O_2_, 5% CO_2_. Where indicated, experiments were performed with a HEPES-buffered aCSF containing (in mM): 114 NaCl, 22 Na-gluconate, 12.5 HEPES, 7.5 Na-HEPES, 5 dextrose, 2.7 KCl, 2 CaCl_2_, 1 MgCl_2_, and 1 NaH_2_PO_4_. pH was adjusted to 7.40 with NaOH, and the solution was gassed with 100% O_2_. The bath was maintained at 34 **°**C for all experiments and tetrodotoxin (TTX) was included in recording solutions to eliminate any possible polysynaptic effects of GABA agonists.

Experiments were performed during ZT 2–8 for ‘day’ mice and ZT 12–18 for ‘night’ mice. Cl-Sensor fluorescent imaging was performed using epifluorescent methodology similar to that described by Friedel *et al*.^69^. Excitation light was supplied by a monochrometer (Polychrome IV; Till Photonics) providing 10 nm bandwidth output. Excitation at 500 nm preceded excitation at 436 nm to promote Cl-Sensor photostability^69^. Excitation duration ranged from 20 to 200 ms, with the excitation at 500 nm 1.5 times longer than that at 436 nm in order to obtain similar intensity values. The fluorescent signal passed through a double bandpass emission filter [470(24) + 535(30) nm] (Chroma Technology Corporation). Imaging was performed with an upright fluorescent microscope and a 63x water-immersion objective, NA 0.90 (Leica). Images were acquired with a charge-coupled device camera (CCD, ORCA-ER 12 bit level; Hamamatsu). Camera gain was set to 100, and binning was 4 × 4. Equipment control and image processing were performed with Metafluor software (Molecular Devices). Regions of interest (ROI) were defined around neuronal soma, and a dim region of the field of view was selected as background. Background values were subtracted for each wavelength independently. Because the YFP moiety of Cl-Sensor is quenched by Cl^−^ ions, we choose to plot the emission following 436 nm excitation over that at 500 nm excitation so that R_Cl_ would be a proxy for [Cl^−^]_i_, with a higher ratio indicating higher [Cl^−^]_i_.

When sampling at 2 and 5 second intervals, an exposure-dependent increase of R_Cl_ was observed, most likely due to an accumulation of Cl-Sensor’s YFP moiety in an inactivated state^69^. To correct for this instability, we fit our data with a single-exponential function, and subtracted the time-dependent component. Despite this correction, we observed that steady-state R_Cl_ remained sensitive to exposure duration. We corrected for this exposure-dependent trend in each condition (AVP+ day, AVP+ night, VIP+ day, VIP+ night) independently in order to avoid any assumptions about the similarities of baseline R_Cl_ across conditions. All values were adjusted to a 500 nm exposure of 100 ms.

### Calibration of Cl-Sensor and estimation of [Cl^−^]_i_

In order to relate R_Cl_ to [Cl^−^]_i_, it was necessary to construct a calibration curve. Cl-Sensor was calibrated using a 0 mM Cl^−^ solution consisting of (in mM): 120 Na-gluconate, 26 NaHCO_3_, 11 dextrose, 2.7 K-gluconate, 2 Ca-gluconate, 1 MgSO_4_, and 1 NaH_2_PO_4_, saturated with 95% O_2_, 5% CO_2_. This solution was mixed with aCSF to produce solutions of 0, 4, 20, 40, 60, and 80, and 123 mM Cl^−^. 50 μM β-escin was added to all calibration solutions to permeabilize cells. AVP::Cl-Sensor and VIP::Cl-Sensor day and night-entrained mice were used for analysis. R_Cl_ values were corrected for exposure as discussed above. Average steady-state R_Cl_ was plotted against Cl^−^ concentration (Supplementary Fig. 1) and calibration data was fit with the following logistic dose-response sigmoidal curve:

1 $${R}_{Cl}={R}_{{\rm{\max }}}+\frac{{R}_{{\rm{\min }}}+{R}_{{\rm{\max }}}}{1+{(\frac{{[{\mathrm{Cl}}^{-}]}_{i}}{{K}_{d}})}^{p}}$$

Where *R*_*Cl*_ is the fluorescence intensity ratio (F_436_/F_500_) for chloride, *K*_*d*_ is the dissociation constant for Cl^−^ binding, *R*_*min*_ and *R*_*max*_ are the minimum and maximum asymptotic values of *R*_*Cl*_, and *p* is the Hill coefficient^33^. To obtain estimates of [Cl^−^]_i_, we re-arranged the equation for [Cl^−^]_i_:

2 $${[C{l}^{-}]}_{i}={K}_{d}\times {(\frac{{R}_{{\rm{\min }}}-{R}_{{\rm{\max }}}}{{R}_{Cl}-{R}_{{\rm{\max }}}})}^{\frac{1}{p}}$$

After fitting the curve, we obtained the following values: *K*_*d*_ = 108.8 mM, *R*_*min*_ = 0.98, *R*_*max*_ = 2.92 and *p* = 2.91 (Supplementary Fig. 1). Our *K*_*d*_ is considerably higher than previously-reported values^33, 70, 71^. We also observed substantial variability in R_Cl_ at specific Cl^−^ concentrations between calibration experiments. Furthermore, R_Cl_ was fairly non-linear in our range of operation (see Supplementary Fig. 1; our R_Cl_ values were generally between 1.0 and 1.3). For these reasons, we elected to leave fluorescent measurements in values of R_Cl_ instead of converting them into estimates of [Cl^−^]_i_ in subsequent analysis.

### Perforated-patch electrophysiology

Brain slices were prepared as above. Recordings were performed with an Axopatch-1D amplifier (Axon Instruments), filtered at 2 kHz, digitized at 5 kHz, and acquired with Patchmaster v5.3 (HEKA Elektronik). Pipette solution contained (in mM): 120 KCl, 20 K-gluconate, 15 HEPES, 2 NaCl, 1 EGTA and either 0.2 of Lucifer Yellow or Texas Red. pH was adjusted to 7.26 with KOH. Gramicidin (Sigma) was dissolved in DMSO to a concentration of 50 mg/mL, aliquoted, and frozen. Before an experiment, this stock solution was diluted to a final pipette concentration of 30–100 μg/mL. A drop of gramicidin-free pipette solution was first applied to the backend of the pipette. After capillary action filled the pipette tip, the pipette was back-filled with the gramicidin-containing solution moments before submersion into the recording chamber. After gigaseal formation, series resistance (R_s_) was monitored with a −5 mV voltage step to monitor the progress of perforation. Only recordings with a R_s_ less than 100 MΩ were used for experiments. Cells were voltage clamped at −60 mV and cells with holding currents less than −30 pA were discarded.

E_GABA_ was determined using voltage ramp protocols. Every 10 seconds, a 400 ms voltage ramp protocol (ΔV ≅ 60 mV) was executed 100 ms after puff-application of 1 mM GABA. A current trace recorded in the absence of GABA was subtracted from currents obtained in the presence of GABA. The subtracted current trace was then plotted against the ramp command potential, and E_GABA_ was recorded as the x-intercept. E_GABA_ was not corrected for R_s_ or liquid junction potentials.

### Drugs

All drugs used in this study were acquired from Tocris Bioscience. Bumetanide and VU0240551 were dissolved in DMSO, stored as 10 mM stock solutions, and applied through the bath at 10 μM. A 100 mM stock of isoguvacine in water was diluted in aCSF to 1 mM and focally applied (5 psi) through a micropipette connected to a Picospritzer (General Valve Corporation).

### Statistics and analysis

Igor Pro (Version 6.22 A; Wavemetrics) was used for plotting, curve-fitting and data analysis. Data are presented as the mean ± standard error. For imaging experiments, generalized estimating equations (GEE) were used to determine statistical significance^72^. GEE models are similar to ANOVA and general linear models in that they estimate a mean response, except standard errors are adjusted for clustered or correlated measurements that originate from multiple observations made from the same brain slice. Therefore, each brain slice is treated as an independent measure, while the ROIs influence the average and standard error of each experiment. GEE test statistics are based on chi-square or z-statistics rather than F- and t-distributions. When comparing drug effects between HEPES-buffered and bicarbonate-buffered solutions, we formed z-statistics equal to the difference between the estimated effects from each separate GEE model (one for each solution) divided by the standard error of the difference. Significance level was Bonferroni-adjusted for these comparisons. For electrophysiology experiments, we used the Student’s *t*-test. For all tests, a *p*-value less than 0.05 was considered to be statistically significant.

### Data availability

The datasets generated during and/or analyzed during the current study are available from the corresponding author on reasonable request.

## Electronic supplementary material

Supplmentary Data
