# Supplementary material for: Intracellular Chloride Regulation in AVP+ and VIP+ Neurons of the Suprachiasmatic Nucleus
Source: Sci Rep. 2017 Aug 31;7:10226. doi: 10.1038/s41598-017-09778-x (PMC5579040; doi:10.1038/s41598-017-09778-x)
Supplement: Supplementary file 1 — Supplmentary Data [file 41598_2017_9778_MOESM1_ESM.pdf]

INTRACELLULAR CHLORIDE REGULATION IN AVP+ AND VIP+ NEURONS OF THE SUPRACHIASMATIC NUCLEUS

Nathan J. Klett[1,2] and Charles N. Allen*[2,3]

[1]Neuroscience Graduate Program, [2]Oregon Institute for Occupational Health Sciences, [3]Department of Behavioral Neuroscience, School of Medicine, Oregon Health & Science University,
Portland, OR 97239

*To whom correspondence should be addressed.
Charles N. Allen, PhD
Oregon Health and Science University
Mail code L606
3181 S.W. Sam Jackson Park Road
Portland, OR 97239-3098
Tel: 503-494-2507
Fax: 503-494-6831
E-mail: allenc@ohsu.edu

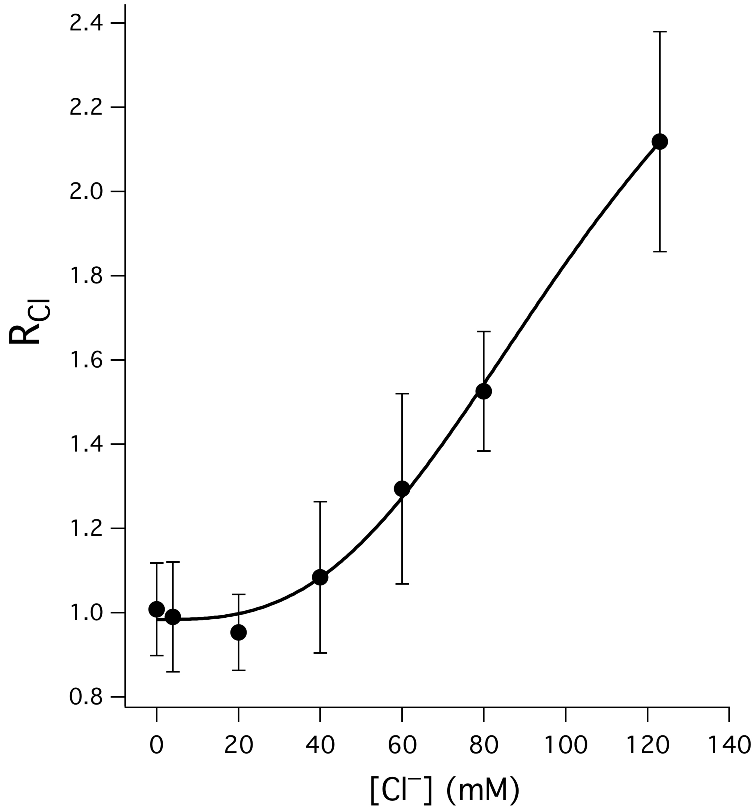

Supplementary Figure 1: Calibration curve of Cl^- Sensor in brain slices

Points are averages ± standard error from multiple experiments. Data were fit with a logistic dose-response sigmoidal curve, modeled after the Hill equation. Curve fitting yielded the following values: K_d = 108.8 mM, R_{min} = 0.98 R_{max} = 2.92 and p = 2.91.

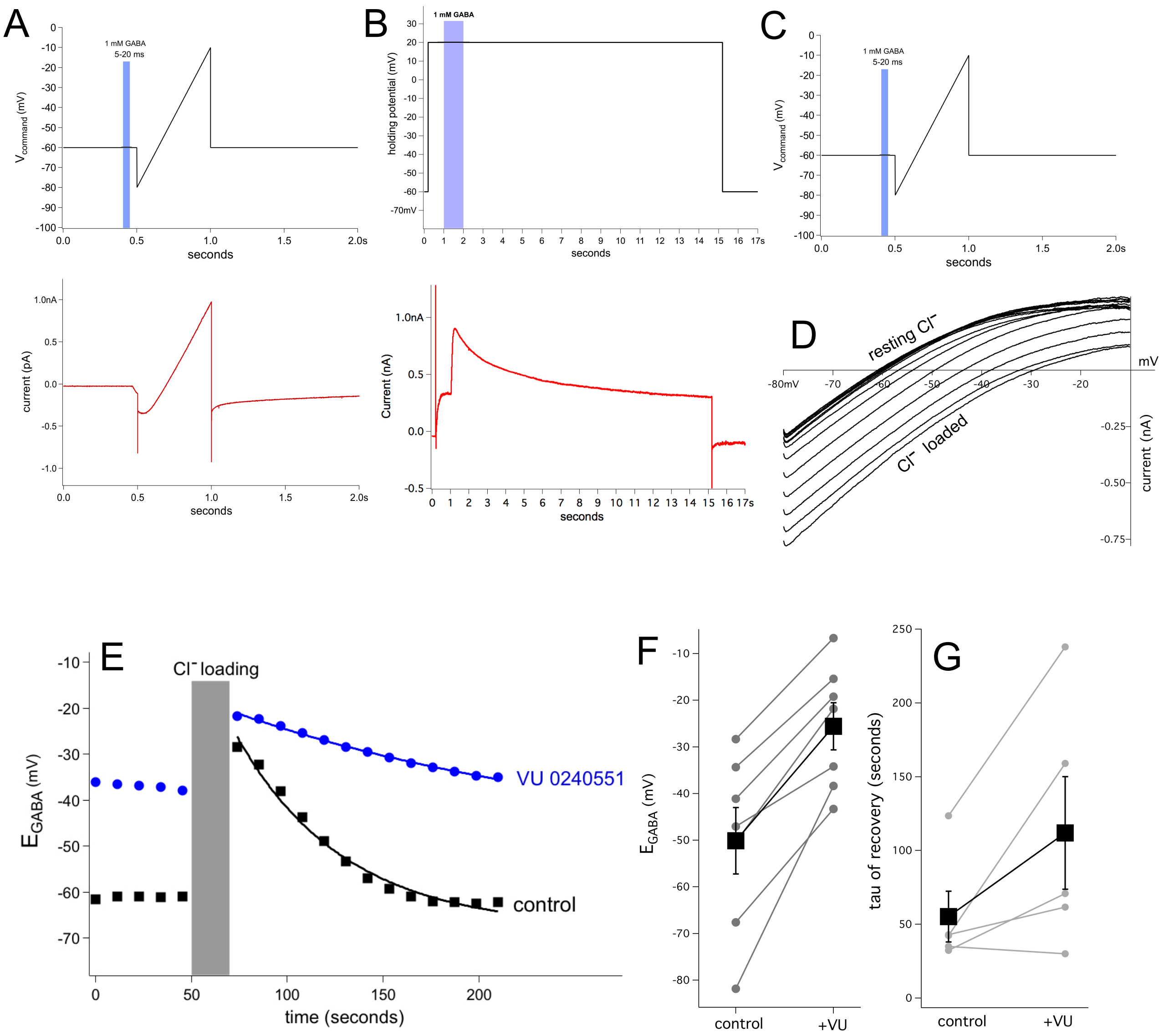

Supplementary Figure 2: The KCCs regulate $[Cl^{-}]_i$ in rat SCN neurons.
(A) Voltage ramp protocol (top) and example current trace (bottom) used to determine resting E_{GABA}. (B) Chloride loading protocol (top) and resultant current trace (bottom). (C) After the loading event, voltage ramp protocols were used again to monitor the recovery of E_{GABA}. (D) IV plots derived from the voltage ramp protocols recorded approximately every 10 seconds, before and after the loading event. Following the chloride loading event, E_{GABA} is depolarized and subsequently recovers to its control value. (E) Example experiment showing the recovery of the GABAergic reversal potential after the Cl^{-} loading protocol. E_{GABA} is plotted against time and fit with a monoexponential function before (τ = 56.2 s, black squares) and after the addition of 20 μM VU (τ = 197.9 s, blue circles). VU increased E_{GABA} (F, $p < 0.001$, paired *t*-test) and slowed the timecourse of recovery (G, $p < 0.05$, paired *t*-test). Cells were selected from the ventral SCN during the day.

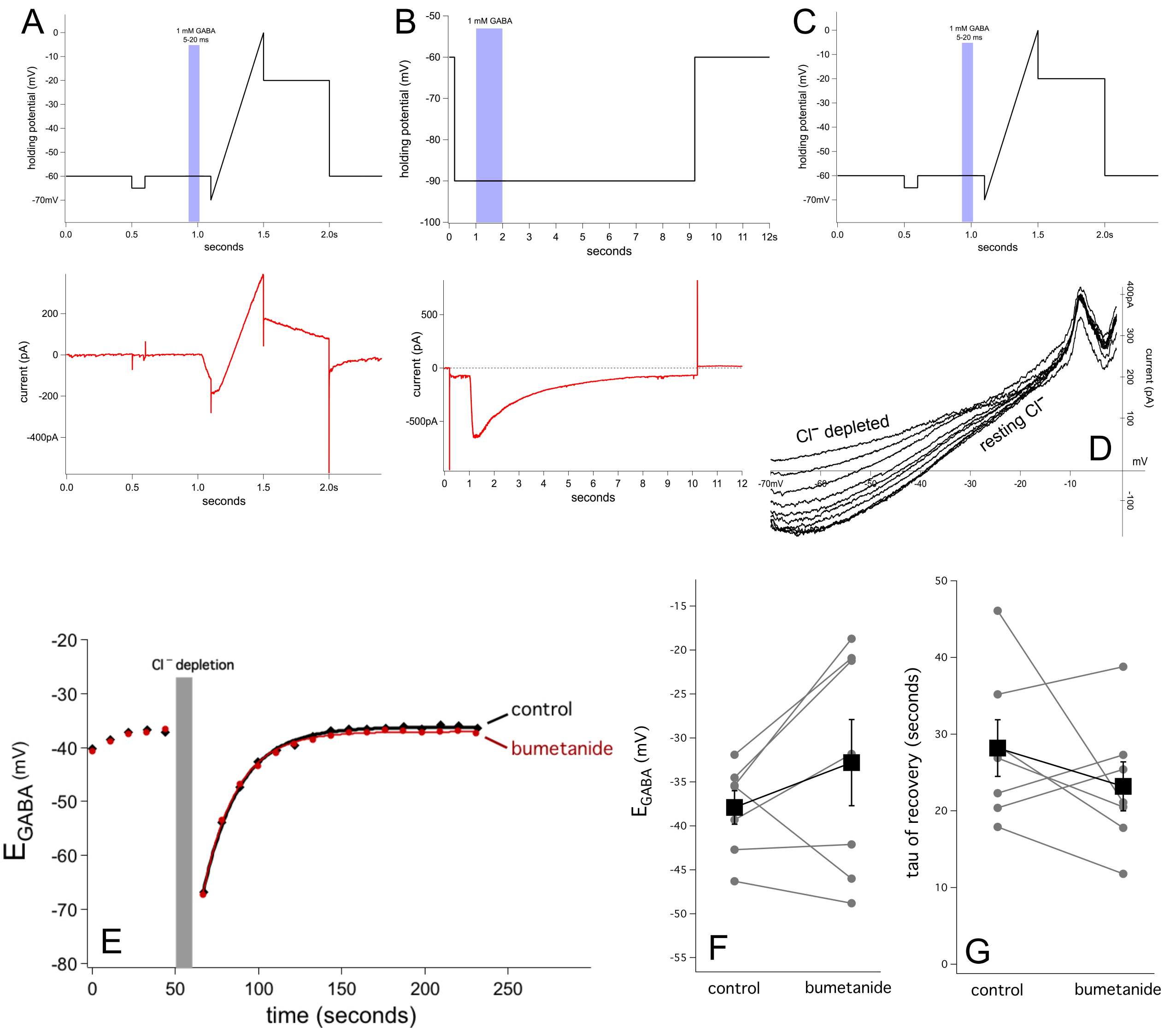

Supplementary Figure 3: NKCC1 does not contribute to resting $[Cl^-]_i$ in rat SCN neurons.
(A) Voltage ramp protocol (top) and example current trace (bottom) used to determine resting E_{GABA}. (B) Chloride depletion protocol (top) and resultant current trace (bottom). The cell was hyperpolarized in the presence of GABA in order to drive Cl^- efflux. (C) After the depletion event, voltage ramp protocols were again used to monitor the recovery of E_{GABA}. (D) IV plots derived from voltage ramp protocols recorded every 10 seconds, before and after the chloride depletion event. Following the chloride depletion event, E_{GABA} is hyperpolarized and subsequently returns to its control value. (E) E_{GABA} plotted against time showing the recovery of E_{GABA} after the chloride depletion protocol. E_{GABA} values are fit with a monoexponential function before (τ = 21.9 s) and after the application of bumetanide (τ = 20.0 s). Bumetanide caused no significant change in either E_{GABA} (F) or the timecourse of recovery after depletion (G). Cells were selected from the dorsal SCN during the night.
